# A Hydrolyzed Soybean Protein Enhances Oxidative Stress Resistance in *C. elegans* and Modulates Gut–Immune Axis in BALB/c Mice

**DOI:** 10.3390/antiox14060689

**Published:** 2025-06-05

**Authors:** Jun Liu, Yansheng Zhao, Fei Leng, Xiang Xiao, Weibo Jiang, Shuntang Guo

**Affiliations:** 1 College of Food Science & Nutritional Engineering, China Agricultural University, Beijing 100083, China; liujun@yuwangcn.com; 2 Shandong Yuwang Ecological Food Industry Co., Ltd., Yucheng 251200, China; zhaoys@ujs.edu.cn; 3 School of Food and Biological Engineering, Jiangsu University, Zhenjiang 212013, China; leng5364118@163.com (F.L.); xiaoxiang1@aliyun.com (X.X.)

**Keywords:** soybean protein, oxidative stress, immune function, gut microbiota

## Abstract

Soy protein isolate (SPI) is a high-purity protein from defatted soybeans, providing emulsifying and gelling functions for plant-based foods and supplements. Hydrolysis can facilitate the production of bioactive small-molecule proteins or peptides with potential functional applications. In this study, 20% hydrolyzed soy protein (20% HSP) was prepared from SPI, and the effects of 20% HSP and SPI on alleviating oxidative stress in *Caenorhabditis elegans* (*C. elegans*) and regulating immune–gut microbiota in cyclophosphamide (CTX)-induced immunocompromised BALB/c mice were investigated. In *C. elegans*, both SPI and 20% HSP (300 μg/mL) enhanced locomotive activities, including body bending and head thrashing, and improved oxidative stress resistance under high glucose conditions. This improvement was mediated by increased antioxidant enzyme activities (SOD, CAT, and GSH-Px), while malondialdehyde (MDA) content was reduced by 60.15% and 82.28%, respectively. Both of them can also significantly extend the lifespan of normal *C. elegans* and paraquat-induced oxidative stress models by inhibiting lipofuscin accumulation. This effect was mediated through upregulation of *daf-16* and suppression of *daf-2* and *akt-1* expression. In immunocompromised mice, 20% HSP alleviated CTX-induced immune dysfunction by increasing peripheral white blood cells and lymphocytes, attenuating thymic atrophy, and reducing hepatic oxidative stress via MDA inhibition. Gut microbiota analysis revealed that 20% HSP restored microbial balance by suppressing *Escherichia-Shigella* and enriching beneficial genera, like *Psychrobacter*. These findings highlight 20% HSP and SPI’s conserved anti-aging mechanisms via *daf-16* activation in *C. elegans* and immune–gut modulation in mice, positioning them as plant-derived nutraceuticals targeting oxidative stress and immune dysregulation.

## 1. Introduction

Supplementing bioactive antioxidant substances and enhancing antioxidant capacity to maintain homeostasis may support overall health [1]. Over the past two decades, plant-based diets have gained attention for their rich content of natural antioxidants [2,3]. Plants and their derivatives, including fruits, vegetables, grains, and processed foods or beverages, are widely acknowledged for their medicinal properties and therapeutic potential, particularly in combating oxidative stress and inflammation [4]. Epidemiological, clinical, and nutritional studies have also demonstrated that dietary plant extracts improve human health by enhancing antioxidant capacity and resistance to oxidative stress [5,6]. The association between dietary functional factors and longevity is increasingly well-established [7,8]. Ginger extracts [9], saponins [10], and soybean protein [11], rich in polyphenols and bioactive proteins, demonstrate antioxidative properties capable of mitigating oxidative stress and extending lifespan across multiple experimental models [12].

As a globally cultivated crop, soybean supplies all essential amino acids required by humans and exhibits high nutritional value [13]. It was found that soybean isoflavones could extend the lifespan of *Drosophila* by enhancing endogenous antioxidant gene expression through transcriptional upregulation [14]. Soy protein further demonstrates preventive potential against chronic diseases, including obesity, cardiovascular disease, insulin resistance/type 2 diabetes [15], and immune dysregulation [16]. Epidemiological evidence indicated that daily consumption of 25 g soybean protein could lower cardiovascular and cerebrovascular diseases risk, positioning it as a strategic dietary intervention for health promotion [17]. Soybean protein has also become one of the best protein sources in residents’ dietary nutrition [18].

Hydrolyzed soybean protein was produced from a soybean protein isolate (SPI) in which either the glycinin or β-conglycinin fractions had been selectively hydrolyzed. It has been found that soybean protein hydrolysates possess high nutritional value and antioxidant properties in the food industry, as well as potential health benefits. The immunomodulatory and anticancer activities of food-derived protein hydrolysates or peptides are related to the amino acid composition, sequence, and length [19]. Additionally, hydrolyzed soybean protein or peptide exhibited a variety of physiological functions [20], such as antioxidant [21], anti-hypertension [22], and anti-cholesterol [23], etc. The consumption of plant proteins and their hydrolyzed forms has been considered a novel strategy for the prevention or treatment of metabolic syndrome and related diseases [24]. A study by Gallyas et al. indicated that bioactive peptides purified from soybean protein isolate enhanced the antioxidant capacity of *C. elegans* and extended their lifespan under oxidative stress [25]. Compared with intact proteins, hydrolyzed soybean proteins may exhibit enhanced bioactivity, which can be attributed to their lower molecular weight and the release of bioactive peptides [26,27]. Notably, low-molecular-weight proteins or peptides demonstrate potential in enhancing immune function and could be applied in the management of immune-related conditions [28,29]. Recent studies demonstrate that enzymatic hydrolysis and fermentation enhance plant proteins’ functional and therapeutic properties by addressing their dependence on inherent properties, anti-nutrients, and digestion for functionality and bioavailability [30]. Therefore, investigating the bioactivity of hydrolyzed soybean proteins holds significant importance for developing high-value functional foods with enhanced nutritional and physiological benefits.

*Caenorhabditis elegans (C. elegans)*, a nematode species, is widely recognized as an ideal model organism in ageing research owing to its compact size, short lifespan, rapid reproductive cycle, high fecundity, optical transparency, ease of observation, and compatibility with high-throughput analyses [5]. Furthermore, *C. elegans* possesses structurally and functionally homologous neuronal, intestinal, and muscle cells essential for neural signaling and metabolic regulation, establishing it as a robust model for studying age-related pathologies, such as neurodegeneration. Notably, its genome contains evolutionarily conserved homologs of approximately two-thirds of human disease-associated genes [31], a feature that has established *C. elegans* as a model system for investigating antioxidant mechanisms, anti-aging pathways, and drug screening over the past several decades.

Furthermore, the gut is considered a vital immune organ in the human body, with the gut microbiota playing a significant role in the regulation of intestinal homeostasis [32,33], thus the immunomodulatory effects of food bioactivity components could be closely linked to their interactions with the gut microbiota [34]. It has been reported that some immunomodulatory peptides can alter the composition and function of the gut microbiota [28], while the mechanism could be different due to the source, structure, and characteristics of proteins and peptides [35].

Therefore, in this work, a hydrolyzed soybean protein (HSP) with a 20% degree of hydrolysis (20% HSP) was prepared from SPI. The antioxidant activities and anti-aging indicators were subsequently evaluated using *C. elegans* model to investigate the beneficial effects of HSP. Then the immunomodulatory activity was investigated using a BALB/c mouse model. To simulate chronic immunosuppression, mice received intraperitoneal injections of cyclophosphamide (CTX) every other day throughout the experimental period. The effects of HSP on immune function and gut microbiota were then analyzed to assess its potential as an immunomodulator in functional foods.

## 2. Materials and Methods

### 2.1. Chemicals and Reagents

The SPI was supplied by Shandong Yuwang Ecological Food Industry Co., Ltd. (Dezhou, China). HSP was prepared from SPI via enzymatic hydrolysis as raw material [36]. The degree of hydrolysis of HSP was determined by the trichloroacetic acid soluble nitrogen (SN-TCA) method [37], and the value was nearly 20%; thus, the sample was designated as 20% HSP. The electrophoretic analysis was performed, and the results are shown in Figure S1.

*C. elegans* and *Escherichia coli* OP50 were originally obtained from the Laboratory of Nutrition and Health, School of Food and Biological Engineering, Jiangsu University (Zhenjiang, China). The detection kits for superoxide dismutase (SOD) activity, catalase (CAT) activity, glutathione peroxidase (GSH-Px) activity, and malondialdehyde (MDA) content were purchased from Nanjing Jiancheng Bioengineering Institute (Nanjing, China). The qRT-PCR reagent was purchased from Takara Biomedical Technology (Dalian) Co., Ltd. (Dalian, China) 2-Deoxy5-fluorouracil nucleoside (FUDR) and methyl violet extract were purchased from Shanghai Aladdin Bio-chem Technology Co., Ltd. (Shanghai, China). The detection kits for TNF-α, IFN-γ, IL-2, IL-4, IL-6, IgM, and IgG, etc. were purchased from Beyotime Biotech Inc. (Shanghai, China). Normal saline and CTX were from Jiangsu Hengrui Medicine Co., Ltd. (Lianyungang, China). Other biochemical reagents were purchased from Sinopharm Group Shanghai Chemical Reagent Co, Ltd. (Shanghai, China).

### 2.2. Determination of the Degree of Hydrolysis of Proteins

The degree of hydrolysis of hydrolyzed soy protein was determined according to the method measured in our previous study [36]. SPI was dissolved in deionized water to prepare a 5% solution. Alkaline protease was added at an enzyme-to-substrate (E/S) ratio of 10% (*w*/*w*). The hydrolysis reaction was conducted at 55 °C for 400 min in a temperature-controlled water bath, with the pH value maintained at 10.0 ± 0.1 throughout the process. The degree of hydrolysis (DH) was calculated according to the following formula.

$$\mathrm{DH}/\;(\%)=\frac{B\;\times \;N}{\alpha \;\times \;m\;\times \;h}\;\times \;100$$

In the formula: B represents the amount of alkali consumed, mL; N represents the molar concentration of alkali, mol/mL; α represents the average degree of dissociation of amino groups. When the pH is 10 and the temperature is 55 °C, α = 0.44; m represents the mass of the protein subjected to enzymatic hydrolysis, in g; h represents the number of mmol of peptide bonds per gram of protein substrate. For the soy protein isolate, h = 8.38 (mmol/g protein).

### 2.3. Culture, Physiological Indexes, and Oxidative Stress Analysis of C. elegans

The nematodes were cultured on nematode growth medium (NGM) plates seeded with *E*. *coli* OP50 (OP50) and maintained at 20 °C. Nematodes were synchronized by using NaClO and NaOH according to the method described by Bai et al. [38]. Synchronized L1 nematodes were supplemented with M9 buffer as a blank or SPI and 20% HSP (300 μg/mL) mixed with *E. coli* OP50 coated on the surface of NGM for 48 h from L1 to L4 prior to use.

For body length and width evaluation, nematodes were washed with M9 buffer and grown on another NGM plate. Synchronized L1 nematodes were treated with 300 µg/mL SPI and 20% HSP for 48 h. Approximately 30 nematodes were subsequently selected and added to M9 buffer with a suitable amount of 0.5 mol/L sodium azide. When the nematode’s body stiffened, a coverslip was placed over it. The length and width were measured using segmented line tools on ImageJ 1.52a.

For head thrashing and body bending analysis, synchronized L1 nematodes were treated with 300 µg/mL SPI and 20% HSP for 48 h. The frequencies of head thrashing and body bending of the nematodes were then counted for 60 s in M9 buffer under a microscope. The nematode’s head deviates from its path with each swing, bending the body’s direction with each movement.

The synchronized nematodes were cultured in NGM plates and NGM medium with 5 mmol/L glucose to established oxidative stress model. Following a 48-h treatment with 300 μg/mL SPI or 20% HSP, the nematodes were rinsed thrice with M9 buffer, homogenized via ultrasonic disruption, and centrifuged at 8000 rpm for 3 min to collect the supernatant. The determinations of SOD, CAT, and GSH-PX activities and MDA content were performed according to the assay kits method. The protein concentration was determined according to the Bradford protein concentration test kit.

### 2.4. Lifespan and Lipofuscin Accumulation Analysis of C. elegans

Lifespan assays were performed using the methods described in our previous research [39]. Synchronous nematodes were grown on NGM to the L4 stage, then washed and transferred to Transwell^®^-24-well permeating plates (BD Biosciences, New York, NY, USA) and treated with S-complete or 300 μg/mL SPI and 20% HSP. The experiments were divided into normal group and oxidative stress group induced with paraquat solution. The medium was changed every 2 days, and the survival rate was recorded every other day until all nematodes died. The day of transfer in L4 was defined as day 0. OASIS 2 (https://sbi.postech.ac.kr/oasis2/surv/, accessed on 26 September 2023) was used to analyze the survival of nematodes.

The nematodes were cultured on NGM normal medium and NGM medium containing 5 mmol/L glucose and treated with 300 μg/mL SPI and 20% HSP for 14 days. The images were captured using an inverted fluorescence microscope. Subsequently, the field of light and fluorescence images of lipofuscin were measured and detected, and the content of lipofuscin were analyzed based on the fluorescence intensity.

### 2.5. Quantitative Real-Time PCR (qRT-PCR) Analysis of C. elegans

Total RNA was extracted by a TaKaRa MiniBEST Universal RNA Extraction Kit (TaKaRa Biotechnology (Dalian) Co., Ltd., Dalian, China). cDNA was reverse transcribed using a Takara PrimeScript RT Master Mix Kit (TaKaRa Biotechnology (Dalian) Co., Ltd., Dalian, China). Quantitative real-time fluorescent PCR was performed according to the instructions of the SYBR^®^ Premix Ex Taq™ Kit (TaKaRa Biotechnology (Dalian) Co., Ltd., Dalian, China). Data were processed according to the threshold cycle method of ΔΔCT. The act-1 gene was used as an internal reference. All primers were designed and synthesized by Sangon Biotech (Shanghai) Co., Ltd. (Shanghai, China) according to the standard, and the primer sequence is listed in Table S1.

### 2.6. Treatment Protocols and Establishment of Immunosuppression Models of Mice

**Figure 1:**
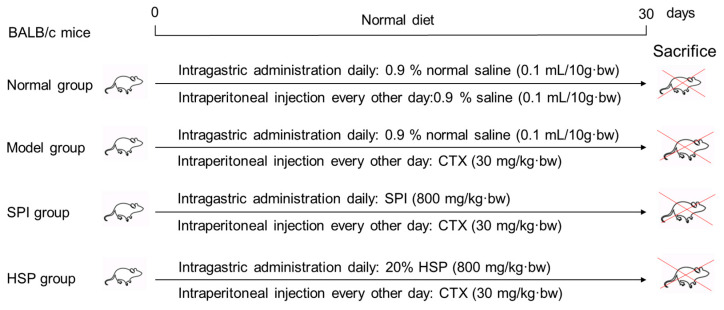
Design of animal experiments.

A total of 60 BALB/c male mice weighing 24 ± 2 g were obtained from Chang Zhou Cavens Laboratory Animal Ltd. (Changzhou, China, license number: SCXK (SU) 2016-0010) and randomly divided into 4 groups with 15 mice per group. Prior to the experiment, the mice were acclimatized in a controlled environment for 3–5 days. The specific treatment protocols are shown in Figure 1 as follows: The immunosuppression model was established by intraperitoneal injection of CTX every other day (30 mg/kg·bw), and the mice in the treatment groups received 800 mg/kg·bw of SPI and 20% HSP per day by intragastric administration, respectively. During the experiment for 30 days, the body hair, weight, behavior, and injection site of mice were observed. Twenty-four hours after the last treatment, the mice were weighed, and the blood of the tail vein was collected with an anticoagulant tube for hemogram determination using a cell analyzer within 12 h. Then, mice were anesthetized by CO_2_ inhalation and sacrificed by cervical dislocation. The animal experiment was approved by the Institutional Animal Care and Use Committee of Jiangsu University (serial number: UJS-IACUC-2020111001).

### 2.7. Measurement of Spleen and Thymus Index and Microscopic Observation of Pathological Sections

The mice were weighed before being sacrificed; then, the thymus and spleen were collected and weighed immediately after removing surface moisture with filter paper. The index of the thymus and spleen were calculated as follows: index (mg/g) = weight of spleen or thymus (mg)/body weight (g).

The intact spleen and thymus were washed with normal saline and fixed in 10% of neutral paraformaldehyde solution. A piece of spleen or thymus tissue was transversally cut and paraffin-embedded after gradient dehydration, and then 3 mm sections were sliced by a Leica RM2235 paraffin slicing machine (Leica Microsystems GmbH, Wetzlar, Germany) and flattened in a warm bath. The tissue sections were placed in an incubator at 37 °C overnight and stored in a refrigerator at 4 °C. Then, the issue sections were deparaffinized, rehydrated, and stained with hematoxylin for 8 min and 0.5% eosin for 1 min. After dehydration and mounting, slides were imaged and observed with a Leica DM6000 B biological microscope (Leica Microsystems GmbH, Wetzlar, Germany).

### 2.8. Determination of Antioxidant Ability of Liver, Cytokine, and Antibody Levels in Mice Serum

The liver was rinsed with ice saline and dried with filter paper. Then the liver was accurately weighed and added with ice saline (M/V = 1:9) to prepare 10% of tissue homogenate. After centrifugation at 4 °C and 1000× *g* for 10 min, the supernatant was obtained and the activities of antioxidant enzymes, including SOD, CAT, and GSH-PX, and the content of peroxide malondialdehyde (MDA) were detected according to the kit instructions.

Fasting for 12 h after the last gavage, the blood was taken and incubated in a clean sterile centrifuge tube at room temperature for 1 h and centrifuged at 3000 r/min for 10 min to obtain the serum supernatant. Then, the levels of tumor necrosis factor-α (TNF-α), interferon-γ (IFN-γ), interleukin-2 (IL-2), interleukin-4 (IL-4), interleukin-6 (IL-6), immunoglobulin M (IgM), and immunoglobulin G (IgG) were detected by ELISA kits, respectively.

### 2.9. Analysis of Intestinal Microbiota in Mice Colon

Microbiota sequencing of mice colon contents was performed by Guangzhou Gene Denovo Biotechnology Co., Ltd. (Guangzhou, China) The DNA of the mice colonic contents was extracted by HiPure Stool DNA Kits, and the DNA quality and purity were detected by a NanoDrop microspectrophotometer (Thermo Fisher Scientific Inc., Waltham, MA, USA). The loading sample volume was 2 μL, and the concentration ranged from 2 ng/μL to 3000 ng/μL. The integrity of nucleic acids was detected by agarose gel electrophoresis, and then, PCR amplification was performed. The 16S rDNA target region of the ribosomal RNA gene was amplified by PCR using primers listed in the Table S2, and the second round of amplification products were purified by AMPure XP Beads and quantified using the ABI StepOnePlus Real-Time PCR System (Life Technologies, Foster City, CA, USA). Then, purified amplicons were pooled in equimolar and paired-end sequenced (PE250) on an Illumina platform (Illumina, Inc., San Diego, CA, USA) according to the standard protocols.

### 2.10. Statistical Analysis

The data for animals from different groups were analyzed by one-way analysis of variance (ANOVA), followed by Tukey HSD (Honestly Significant Difference) test for multiple comparison with GraphPad Prism 9.00 software (La Jolla, CA, USA). *p* < 0.05 indicated a statistically significant difference. The original data of intestinal microbiota was analyzed by an online omics tool, which is MicrobiomeAnalyst 2.0 (https://www.microbiomeanalyst.ca/, accessed on 31 December 2024) [40], and the figures were re-plotted with GraphPad Prism 9.00.

## 3. Results and Discussions

### 3.1. Effects of SPI and 20% HSP on the Growth Index and Locomotive Activities of C. elegans

**Figure 2:**
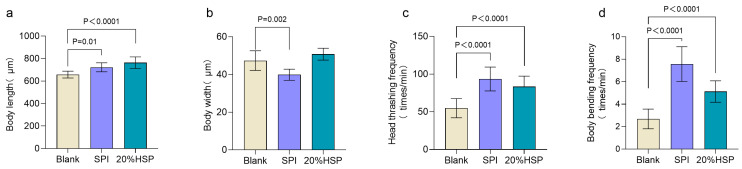
Effects of 20% HSP on growth index and locomotive activities of *C. elegans* ((**a**). Body length; (**b**). Body width; (**c**). Head thrashing frequency; (**d**). Body bending frequency). Bars indicate means ± SD (n = 8 and n = 16, respectively).

The growth and developmental status of nematodes are quantitatively assessed through key morphometric and behavioral parameters, including body length, body width, and locomotor activity. To assess the effects of SPI and 20% HSP on nematode growth, morphometric parameters were quantitatively monitored. During the growth period, nematode body length exhibits progressive elongation, whereas a marked reduction in body length occurs during the senescence phase due to age-related muscle atrophy [41]. In addition, the locomotive ability of nematodes decreases as lifespan increases [42]. As shown in Figure 2a, SPI and 20% HSP increased the body length of nematodes and promoted their growth. In locomotive behavior, body bending and head thrashing are the most sensitive physiological indicators of nematodes [43,44]. The results showed that the body bending frequency decreased significantly. SPI and 20% HSP improved the frequency of nematode head thrashing and body bending. Therefore, the experimental results showed that soybean protein supplementation possessed the function of improving the locomotive capacity of nematodes during senescence.

### 3.2. Effects of SPI and 20% HSP on Oxidative Stress of C. elegans

**Figure 3:**
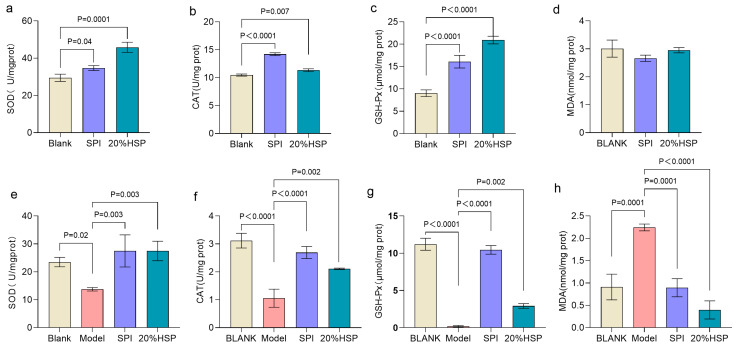
Effects of 20% HSP on SOD, CAT, and GSH-Px activities and MDA content of *C. elegans* ((**a**–**d**): *C. elegans* was under normal conditions; (**e**–**h**): Oxidative stress was induced by high glucose). Bars indicate means ± SD (n = 3).

Excessive accumulation of reactive oxygen species and free radicals in the body can be eliminated by complex enzymatic or non-enzymatic antioxidant defense systems to maintain physiological homeostasis [45,46]. Superoxide dismutase (SOD), catalase (CAT) and glutathione peroxidase (GSH-Px), as the main enzymes protecting tissues from oxidative damage, are crucial indicators to evaluate the antioxidant capacity of the body [47]. As shown in Figure 3a–c, compared to the normal nematodes in the blank group, the SOD, CAT, and GSH-Px activities of nematodes in SPI and 20% HSP group were increased by 17.71%, 35.72%, and 78.28% and 55.52%, 8.28%, and 131.82% respectively, possibly related to the biological role of bioactive peptides in SPI, which undergo gastrointestinal hydrolysis into low-molecular-weight oligopeptides capable of modulating antioxidant defense systems [25]. The level of malondialdehyde (MDA) exhibits a dose-dependent relationship with oxidative damage [48]. As shown in Figure 3d, although MDA concentrations in both the SPI and 20% HSP groups showed a modest reduction compared to the blank control group, the differences were not statistically significant.

It has been reported that high glucose induction can cause oxidative stress in nematodes [49]. To further evaluate the antioxidative potential of SPI and 20% HSP under oxidative stress, a high-glucose-induced oxidative stress model was established in *C*. *elegans*. As shown in Figure 3e–g, SOD, CAT, and GSH-Px activities of nematodes in the model group were significantly decreased (*p* < 0.05), which were 42.50%, 66.09% and 98.27% lower than those in the blank group, respectively. The MDA content of the model group was significantly higher than that of the blank group (*p* < 0.0001) (Figure 3h), increasing from 0.91 mol/mg prot to 2.24 mol/mg prot, indicating that the high-glucose-induced oxidative stress model was successfully validated in this study. When SPI and 20% HSP was administered to high-glucose-induced nematodes, the activity of antioxidant enzymes increased significantly, which were 2.00-fold, 2.55-fold, and 44.95-fold and 2.00-fold, 2.00-fold, and 11.36-fold, respectively, compared to the model group. In addition, the MDA content was significantly decreased from 2.24 mol/mg prot in the model group to 0.89 mol/mg prot in the SPI group and 0.40 mol/mg prot in the 20% HSP group, a decrease of 60.15% and 82.28%. The inhibitory effect of SPI on MDA may be due to the fact that the active peptide components of soybean protein isolate can directly scavenge ROS generated during metabolism or inhibit the intermediate link of lipid peroxidation, thus effectively blocking the process of the oxygen free radical reaction chain [29]. It can be concluded that SPI and 20% HSP enhanced the activity of antioxidant enzymes, thus enhancing the antioxidant capacity of nematodes and resisting the body damage caused by oxidative stress, which may be related to bioactive components, such as soybean protein peptide and amino acid residues [50,51,52].

### 3.3. Effects of SPI and 20% HSP on the Lifespan of C. elegans

**Figure 4:**
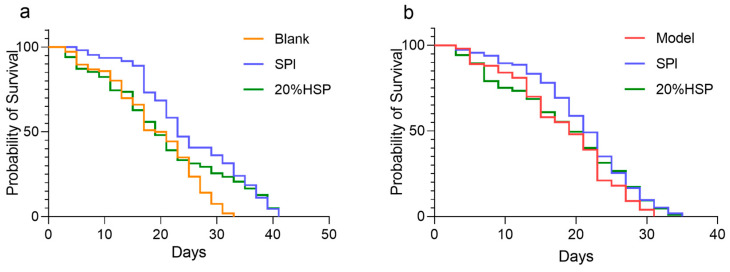
SPI prolonged the survival of *C. elegans* ((**a**). *C. elegans* was under normal conditions; (**b**). Oxidative stress was induced by paraquat). The survival rate was calculated every other day, and OASIS was applied to analyze the differences between groups.

Relevant studies have shown that soybean protein, as an important plant-derived food, has a significant effect on delaying ageing [14]. To determine the effects of SPI and 20% HSP on nematode longevity, lifespan was assessed using the liquid culture method. As illustrated in Figure 4a, the SPI group showed an average lifespan of 24.9 ± 0.8 days, representing a 32.73% increase compared with the blank group (18.8 ± 0.8 days, *p* < 0.0001), indicating that SPI significantly prolonged the nematode lifespan.

The free radicals produced by paraquat can induce oxidative stress damage in nematodes, and the lifespan of nematodes is shortened under oxidative stress [53]. In this experiment, paraquat was used to induce oxidative stress in nematodes. The results suggested that SPI and 20% HSP treatment were protective against oxidative stress. Compared with the model group that was under paraquat-induced oxidative stress (18.10 ± 0.73 days), the average lifespan of nematodes in the SPI group was 20.86 ± 0.69 days, and the SPI and 20% HSP survival curve were significantly shifted to the right. The lifespan of nematodes was extremely significantly prolonged (*p* < 0.005), indicating that SPI and 20% HSP treatment enhanced the resistance of nematodes to oxidative stress, which contributed to the extension of the nematode lifespan and the delay of nematode senescence. Increased stress resistance has been suggested as one of the mechanisms by which phytochemical extracts extend lifespan [54].

### 3.4. Effect of SPI on Lipofuscin Accumulation in C. elegans

**Figure 5:**
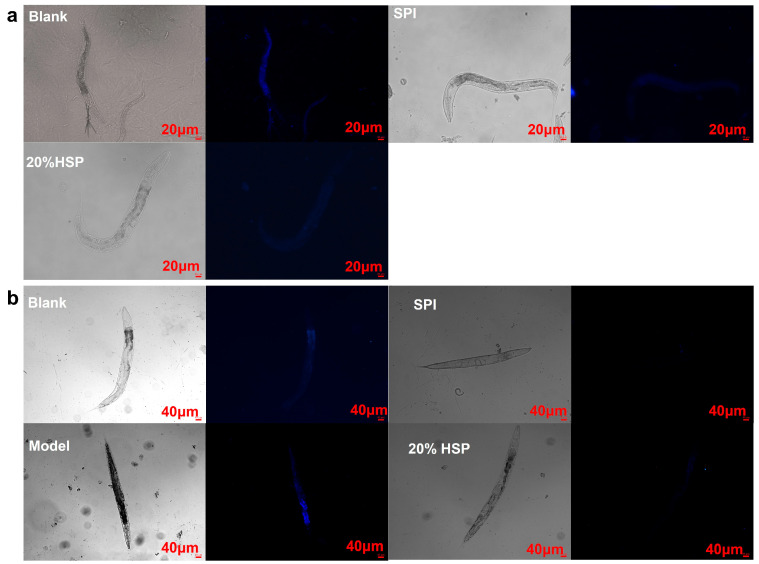
Effects of SPI and 20% HSP on the lipofuscin accumulation of *C. elegans*. ((**a**). *C. elegans* was under normal conditions; (**b**). Oxidative stress was induced by high glucose).

Lipofuscin, a type of self-fluorescent pigment, is an indicator of oxidative stress and ageing in nematodes [43,55,56]. To investigate whether SPI and 20% HSP treatments enhance health span, lipofuscin accumulation in nematodes was quantified using inverted fluorescence microscopy. As shown in Figure 5, compared to the normal nematodes in the blank group, the SPI and 20% HSP groups exhibited reduced lipofuscin-associated blue autofluorescence intensity, demonstrating a significant decrease in age-pigment accumulation (Figure 5a). Lipofuscin accumulation, a hallmark of cellular ageing, is driven by oxidative damage to cellular components and impaired autophagy–lysosomal degradation pathways. These results demonstrate that SPI and 20% HSP treatments significantly reduce age-associated lipofuscin deposition in nematodes, likely through attenuation of ROS-mediated oxidative stress. As shown in Figure 4b, high-glucose-induced oxidative stress markedly enhanced lipofuscin accumulation, whereas SPI and 20% HSP treatments effectively suppressed this phenotype. This observation suggested that the lifespan extension mediated by these treatments may stem from enhanced oxidative stress resistance. Collectively, the significant reduction in age-pigment accumulation reflects improved health span in nematodes, establishing SPI and 20% HSP as potent anti-ageing interventions.

### 3.5. Effects of SPI and 20% HSP on the Expression of Aging-Related Genes

**Figure 6:**
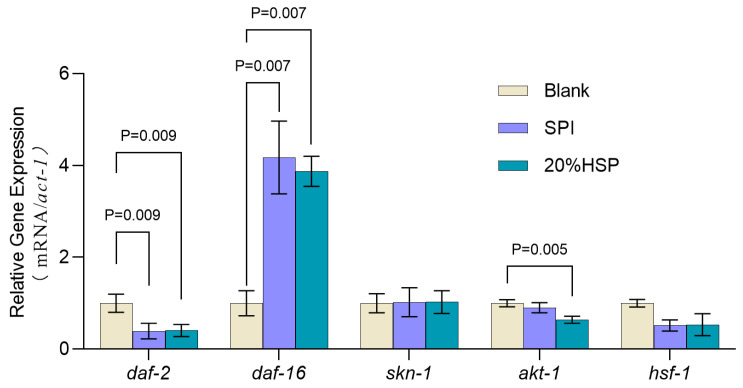
Expressions of antioxidant-related genes of *C. elegans*. *C. elegans* was treated with SPI and 20% HSP at 300 μg/mL for 48 h from L1 to L4, and gene expression was analyzed by qRT-PCR. Data are expressed as means ± SEM, n = 3.

To further explore the anti-ageing mechanism of SPI and 20% HSP, the expressions of longevity-associated genes were measured. As reported, the insulin signaling pathway plays an important regulatory role in nematode development, metabolism, and ageing and is highly conserved and the most detailed lifespan regulatory link affecting animal ageing [57]. daf-2 is the only member of the insulin receptor family expressed in nematodes. As a major upstream regulator of the IIS pathway, daf-2 inhibits daf-16 nuclear translocation for transcriptional regulation, thereby affecting the expression of ageing-related genes [58,59]. It has been reported that loss of daf-2 function can extend the lifespan of nematodes threefold [60]. As shown in Figure 6, the transcript level of daf-2 significantly decreased after SPI and 20% HSP treatment (*p* < 0.05), indicating that the daf-2 transcription factor played a key role in the insulin signaling pathway in which SPI and 20% HSP delayed the senescence of nematodes. Importantly, the insulin signaling pathway regulates the activity of three transcription factors, daf-16, hsf-1, and skn-1, thereby inducing the expression of a number of genes involved in stress response, homeostasis regulation, and metabolism. The effect of SPI and 20% HSP on the mRNA levels of the three transcription factors was analyzed by qRT-PCR. The results showed that SPI and 20% HSP treated nematodes exhibited increased daf-16 transcript levels, decreased hsf-1 expression, and unchanged skn-1 mRNA levels. Therefore, these findings suggested that the lifespan-extending efficacy of SPI and 20% HSP in nematodes may depend on the daf-16 transcriptional activity. Furthermore, as an upstream regulator in the insulin signaling pathway, inhibition of akt-1 transcription levels can significantly extend the lifespan of nematodes. The results showed that the transcript level of akt-1 in nematodes was significantly decreased, indicating that the akt-1 transcription factor played a key role in the lifespan extension of nematodes by 20% HSP.

In conclusion, administration of soybean protein isolate (SPI) and 20% HSP significantly extended nematode lifespan through targeted modulation of the insulin/IGF-1 signaling (IIS) pathway. The analyses revealed a critical regulatory mechanism: both treatments induced a significant downregulation of daf-2, the IIS pathway’s primary receptor homolog, thereby relieving its inhibitory effect on downstream effectors. This suppression of daf-2 transcription triggered a cascade of events, including reduced akt-1 transcript levels, which encodes a serine/threonine kinase responsible for phosphorylating and sequestering DAF-16 in the cytoplasm. Notably, the differential regulation of daf-2 and akt-1 versus terminal transcription factors daf-16, hsf-1, and skn-1 indicated that SPI and 20% HSP exert their anti-aging effects primarily through daf-16-dependent mechanisms rather than broadly activating all IIS downstream targets. This specificity may explain the enhanced stress resistance and delayed senescence observed in treated nematodes.

### 3.6. Effect of HSP on Weight, Behavior, and Physical State of Mice

**Figure 7:**
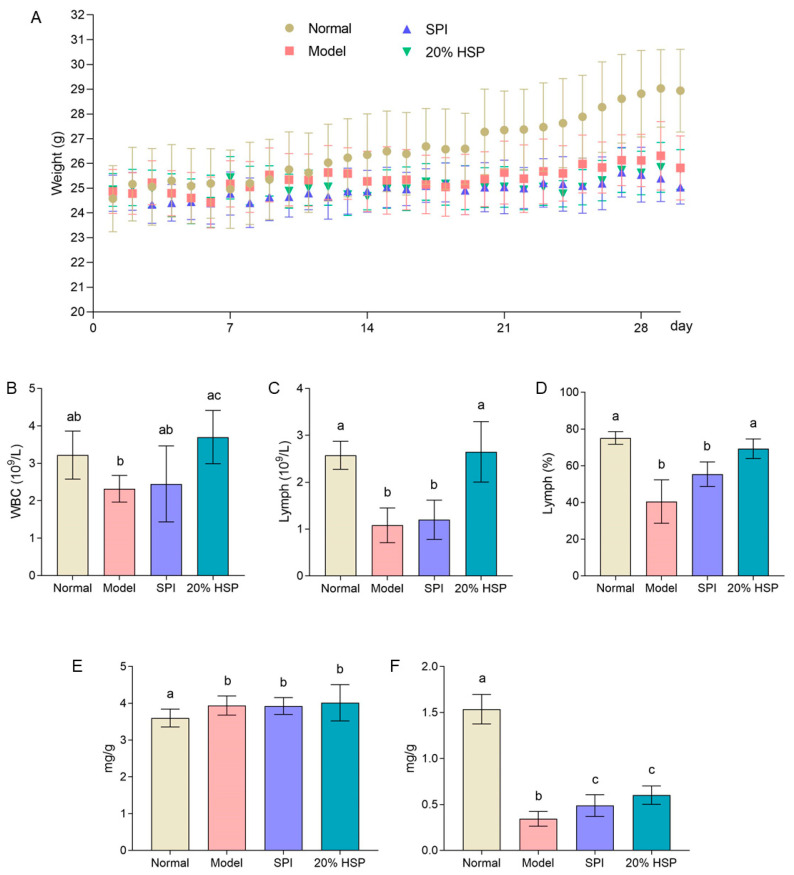
The body weight curve (**A**), hemogram (**B**–**D**), spleen index (**E**), and thymus index (**F**) of mice. Different letters indicate significant differences between groups (*p* < 0.05).

As shown in Figure 7A, the body weight of mice in the normal group was significantly greater than that of CTX-treated mice after two weeks, while minimal differences were observed among the SPI group, HSP group, and model group. By the end of the experiment, the normal mice exhibited an average body weight increase of approximately 15%, whereas the model group, SPI group, and 20% HSP group showed only 5%, 2%, and 4% increases, respectively. These results demonstrate that CTX severely suppressed weight gain in mice, and neither SPI nor 20% HSP effectively counteracted this suppression. Interestingly, as the experiment progressed, the mice in the model group developed significantly disheveled fur following CTX injection, whereas those in the SPI and 20% HSP groups exhibited improved coat luster. Furthermore, during intragastric administration and intraperitoneal injection procedures, mice treated with SPI and 20% HSP demonstrated greater vitality, stronger disease resistance, and more robust limb strength compared to the model group. No signs of infection were observed at any intraperitoneal injection sites across all groups. The above results indicated that administering CTX every other day throughout the experiment exerted a significant inhibitory effect on mice, while protein intake may positively influence physical fitness recovery.

### 3.7. Effect of HSP on Hemogram of Mice

As a critical indicator of immune function, the white blood cell (WBC) count directly reflects changes in the body’s immune status. A decrease in the WBC count indicates a compromised immune system. Lymphocyte (Lymph), key components of adaptive immunity, mediate specific immune responses. Their quantitative reduction may signal impaired immune system functionality, as lymphocyte levels correlate with the body’s capacity to mount effective immune responses [61]. As shown in Figure 7B, the WBC count in the model group exhibited a decreasing trend compared to the normal group, whereas the 20% HSP group demonstrated a significant 59% increase in mean WBC count relative to the model group. Meanwhile, the lymph number and lymph percentage in the model group were much lower than that of the normal group; the intake of SPI could not increase them significantly, while 20% HSP showed improvement ability. Both indicators recovered to similar levels to the normal group. Furthermore, both lymphocyte count (Lymph#) and lymphocyte percentage (Lymph%) in the model group were markedly lower than those in the normal group. While SPI administration failed to significantly elevate these parameters, the 20% HSP intervention restored both indices to levels comparable with the normal group. The results indicated that compared with SPI, 20% HSP could significantly promote the immune function indexes in the hemogram of mice under the condition of immunosuppression caused by CTX. The enhanced immunorestorative capacity of 20% HSP over SPI in CTX-treated mice likely stems from its bioactive proteins or peptides that enable efficient absorption to stimulate hematopoietic differentiation—a mechanism potentially conserved across species as evidenced by HSP’s stronger DAF-16 activation in nematodes.

### 3.8. Effect of HSP on the Spleen and Thymus in Mice

The spleen and thymus are known as key immune organs. As shown in Figure 7E, it is found that the spleen indices of CTX-treated mice including model group, SPI group and 20% HSP group were higher than that of normal mice significantly. This paradoxical increase occurred despite similar absolute spleen weights across groups, primarily attributable to the markedly reduced body weight in CTX-exposed mice. Chronic CTX administration induced moderate spleen index elevation rather than atrophy, potentially indicating compensatory hypertrophy through sustained splenic stress responses. While 20% HSP restored lymphoid parameters, its inability to normalize spleen indices suggests distinct regulatory mechanisms between hematopoietic recovery and organosomatic adaptation. Meanwhile, the thymus indices in CTX-treated mice demonstrated a marked 50% reduction compared to the normal group (*p* < 0.001) (Figure 7F), indicating distinct thymotoxic effect of chronic CTX administration [62]. As a result, all CTX-treated groups could not return to baseline thymic indices post-intervention. Nevertheless, both SPI and 20% HSP supplementation significantly attenuated CTX-induced thymic atrophy, with mean thymus indices in the SPI group and 20% HSP group demonstrating 42% and 75% elevations, respectively, compared to the model group. This showed that SPI and 20% HSP could relieve the severe atrophy of thymus caused by intraperitoneal injection of CXT, although they could not return to normal. This partial restoration indicated that while these protein interventions mitigate thymic involution through potential mechanisms, complete thymic reconstitution requires additional microenvironmental support beyond nutritional supplementation alone, especially under chronic CTX administration.

**Figure 8:**
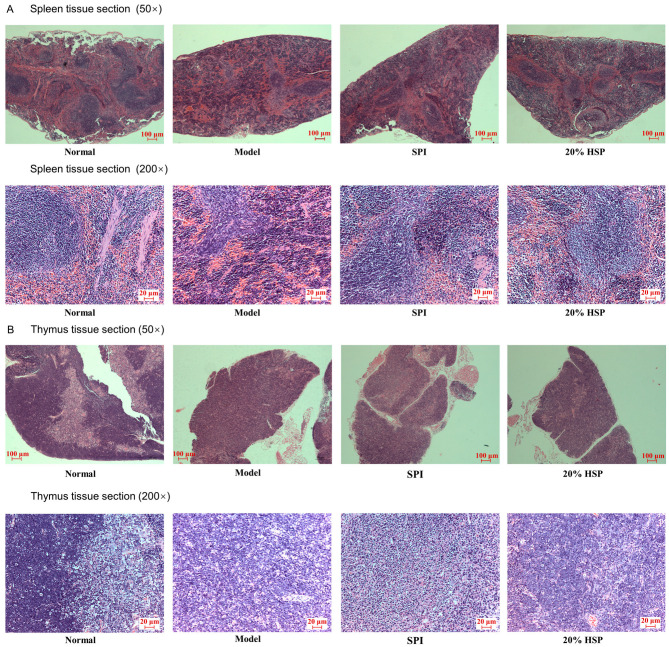
Microphotographs of spleen (**A**) and thymus (**B**) tissue sections of mice (50× and 200×).

Histopathological analysis of H&E-stained spleen and thymus tissue sections was conducted to evaluate CTX-induced immunotoxicity and therapeutic interventions by SPI and 20% HSP. As shown in Figure 8, splenic architecture in normal mice maintained distinct demarcation between red pulp and white pulp. In contrast, CTX-treated model group spleens displayed structural disorganization with red pulp–white pulp boundary effacement (Figure 8B vs. Figure 8A), indicative of CTX-induced lymphoid tissue damage. Meanwhile, histological analysis revealed distinct corticomedullary demarcation in normal murine thymus, whereas the CTX-treated model group exhibited complete corticomedullary junction effacement with a decrease in thymocytes (Figure 8B), confirming CTX-induced thymic histoarchitecture disruption, indicating CTX’s dual toxicity on both central (thymus) and peripheral (spleen) immune organs. The administration of SPI and 20% HSP partially ameliorated CTX-induced histopathological damage. Notably, the 20% HSP group exhibited near-normal splenic architecture with restored red pulp–white pulp demarcation. Thymic sections revealed increased thymocyte density with partial corticomedullary boundary re-establishment, though full recovery was not achieved (Figure 8B). The superior tissue repair capacity of 20% HSP aligns with its previously observed lymphocyte restoration. Twenty percent HSP may reduce oxidative damage to stromal cells, maintaining lymphoid organ microarchitecture. The enhanced DAF-16 activity shown in nematode studies could promote thymic and splenic cell regeneration.

### 3.9. Effect of HSP on Antioxidant Capacity in Mice Liver

**Figure 9:**
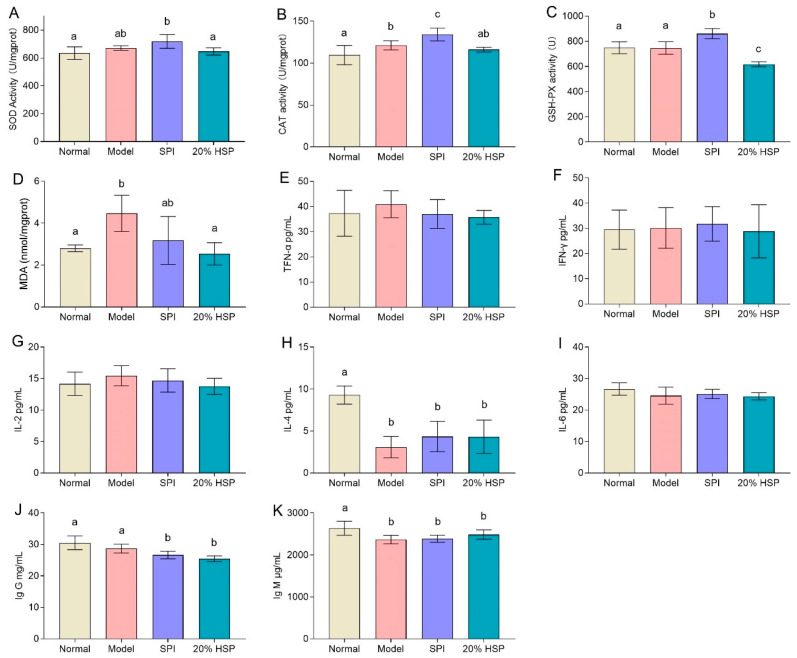
Effect of SPI, HSP, and SPP on the activity of SOD (**A**), CAT (**B**), and GSH-PX (**C**) and the content of MDA (**D**) in mice liver and the levels of TFN-α (**E**), IFN-γ (**F**), IL-2 (**G**), IL-4 (**H**), IL-6 (**I**), Ig G (**J**), and IG M (**K**) in mice serum. Different letters indicate significant differences between groups (*p* < 0.05).

In this study, SOD activities in mice liver showed no significant differences among the normal, model, and 20% HSP groups, whereas the SPI group exhibited an upward trend compared to the normal and 20% HSP groups. The activities of CAT and GSH-PX of mice liver in the SPI group were higher than those in other groups, while 20% HSP failed to enhance the antioxidant enzyme activity (Figure 9A–C). This phenomenon may be attributed to CTX’s atypical oxidative stress induction, which disrupted hepatic oxidative balance without markedly reducing antioxidant enzyme activity. The MDA analysis further supported this interpretation. As a typical carbonyl compound originating from lipid oxidation, MDA is usually used to reflect the degree of oxidative stress [63]. It was shown that the MDA content in the model group increased by 60% compared with that in the normal group, while that in the 20% HSP group decreased by 20% compared with the model group. There was no significant difference between the 20% HSP group and the normal group (Figure 9D). The above results indicated that the activities of antioxidant enzymes in liver were not affected in the immunocompromised mice, indicating the mice might be in a state of high oxidative stress due to the long-term and continuous injection of CTX. Furthermore, 20% HSP significantly inhibited the production of MDA, demonstrating a potential protective effect against oxidative stress and thus helping to alleviate the liver damage caused by CTX.

In summary, these findings demonstrated that chronic CTX exposure induced a state of elevated oxidative stress in immunocompromised mice, characterized by unaltered antioxidant enzyme activity but significantly increased MDA levels. The administration of 20% HSP effectively mitigated this oxidative damage by suppressing MDA production, suggesting its potential as a protective agent against CTX-induced oxidative injury.

### 3.10. Effect of HSP on the Levels of Cytokine and Immunoglobulin in Mice Serum

In general, immunosuppression may increase the risk of inflammation, so serum cytokine analysis can be used to evaluate the likelihood of inflammation or infection in mice. TNF-α, a cytokine playing a central role in inflammation, apoptosis, and immune system development, serves as a key inflammatory marker. Meanwhile, IFN-γ is a key cytokine related to anti-tumor immunity, which protects the host by inhibiting cell proliferation, promoting cancer cell apoptosis and immune response. Interleukins, including IL-2, IL-4, IL-6, and IL-12, exhibit biological activities and play vital roles in mediating cellular immunity, resisting microbial infection, and exerting anti-tumor effects.

As shown in Figure 9E–G,I, there was no significant difference in TNF-α, IFN-γ, IL-2, and IL-6 levels across groups, which indicated that continuous intraperitoneal injection of CTX did not induce significant inflammation in mice, suggesting an absence of major bacterial infections during the experiment. This outcome thus facilitates further research on intestinal microbiota. Additionally, these observations may also reflect CTX’s anti-inflammatory effects as a chemotherapeutic agent.

Figure 9H shows that the IL-4 levels in the model group, SPI group, and HSP group were lower than those in the normal group. Although SPI and HSP intake exhibited an increasing trend in IL-4 compared to the model group, neither significantly increased IL-4 levels. While IL-2, IL-4, and IL-6 are all important immunomodulators, their roles and mechanisms differ within the immune system. IL-4, a type II helper cytokine secreted by T cells, primarily stimulates and activates B-cell and T-cell proliferation. Thus, reduced IL-4 levels may serve as a key indicator of immunosuppression in this mice model. Similar to the IL-4 analysis results, Ig G and Ig M levels exhibited a deceasing trend in the model comparing SPI and 20% HSP groups to the normal group. Neither SPI nor 20% HSP intake restored Ig G or Ig M to normal levels (Figure 9J,K). These findings suggested that CTX suppressed immunoglobulin production, while further supporting the absence of serious infections during the experimental period.

### 3.11. Effect of HSP on Intestinal Microbiota Diversity in Mice

**Figure 10:**
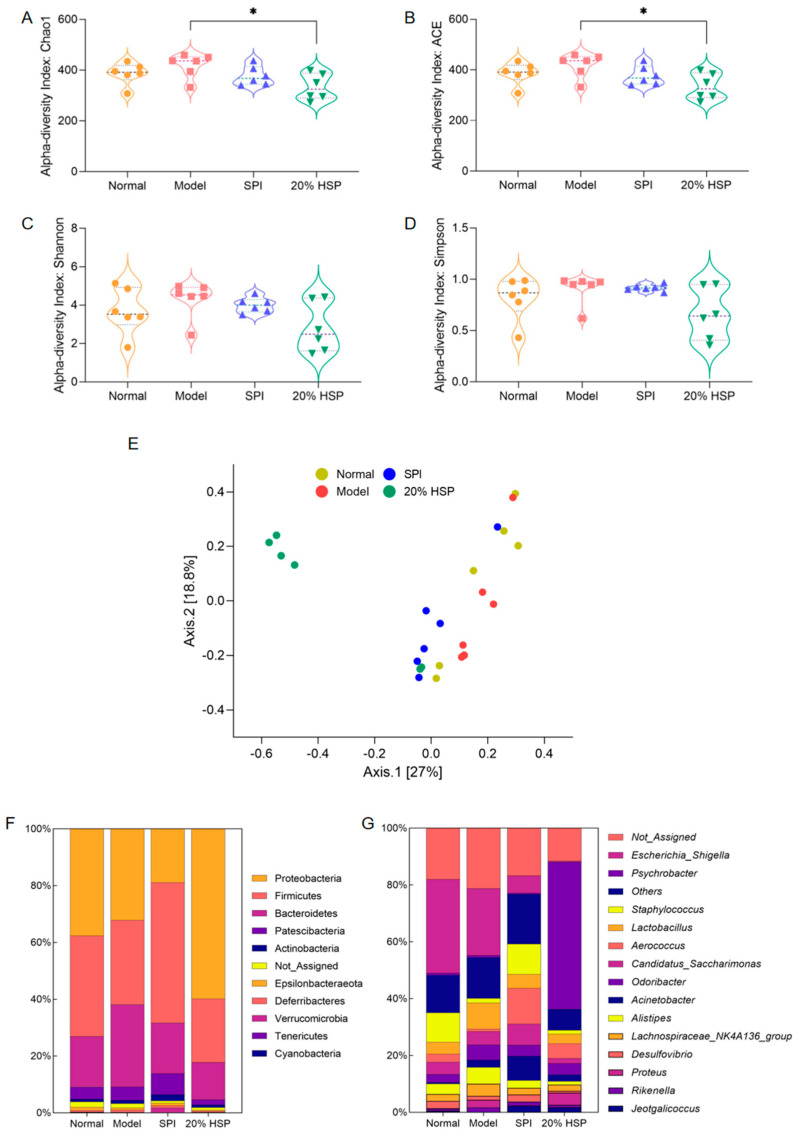
Effect of HSP on Alpha diversity (**A**–**D**), Beta diversity (**E**), abundance percentages at the phylum (**F**) and genus (**G**) of intestinal microbiota in mice colon. “*” indicate significant differences between groups (*p* < 0.05).

Alpha diversity and beta diversity (Figure 10E) were analyzed to investigate CTX-, SPI-, and 20% HSP-induced changes in intestinal microbiota. As shown in Figure 10A–D, alpha diversity indices, including Chao1, Ace, Shannon, and Simpson, showed no significant differences among the four groups, except for lower Chao1 and Ace indices in the 20% HSP group versus the model group. Shannon and Simpson indices revealed a downward trend in the 20% HSP group relative to other groups. This indicated that CTX might not severely alter colonic microbiota diversity, while 20% HSP may reduce the microbiota diversity by inhibiting the proliferation of specific gut bacteria.

Beta diversity results based on principal coordinates analysis (PCoA) aligned with alpha diversity trends. As shown in Figure 10E, samples from most groups formed distinct clusters despite 1 or 2 outliers, with the 20% HSP group exhibiting particularly pronounced separation. It indicated that significant beta diversity differences among groups despite minimal alpha diversity variation, suggesting 20% HSP induces substantial microbiota restructuring. Consequently, deeper microbiota analysis would help to elucidate sample-specific impacts on intestinal microbiota.

### 3.12. Effects of HSP on the Composition of Intestinal Microbiota in Mice

The dominant gut bacteria are closely related to the ecological and functional structure of the microbial community, effectively explaining community structure formation, dynamics, and ecological impact. As shown in Figure 10F, Proteobacteria, Firmicutes, Bacteroidetes, and Patescibacteria were the dominant bacteria phyla in the mouse colon [64], accounting for more than 80% of the total microbiota. Subsequently, the abundance of intestinal microbiota at the genus level is presented in Figure 10G, while comparative changes across groups are visualized in the heatmap (Figure S2). It was found that *Escherichia-Shigella* constituted a major proportion of the microbial community in the normal group, and CTX appeared to inhibit *Escherichia-Shigella* in the model group. Meanwhile, both SPI and 20% HSP suppressed the proliferation of *Escherichia-Shigella*, which was a substantial compositional shift in the gut microbiota. In addition, the SPI group and 20% HSP group exhibited distinct alterations in other bacterial populations. For instance, *Staphylococcus* was suppressed in the model and 20% HSP groups relative to the normal group, while *Aerococcus* proliferation increased in the SPI and 20% HSP groups. In addition, *Psychrobacter* accounted for >50% of the microbiota in the 20% HSP group, which was markedly higher than the <1% observed in other groups.

**Figure 11:**
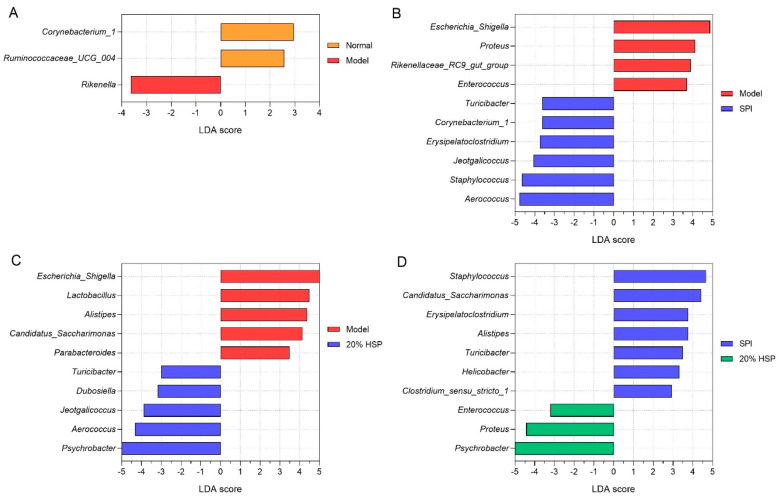
Difference analysis of colonic microbiota. (**A**) LEfSe analysis between the normal and model group; (**B**) LEfSe analysis between the model and SPI group; (**C**) LEfSe analysis between the model and 20% HSP group; (**D**) LEfSe analysis between the SPI and 20% HSP group.

Linear discriminant analysis (LDA) effect size (LEfSe) was employed to analyze intergroup microbiota differences, with results ranked by LDA score. As shown in Figure 11, the Y-axis displays genera exhibiting significant abundance differences (*p* < 0.1), while bar length represents the effect magnitude (LDA score) of each taxon. According to Figure 11A, differentially abundant genera, such as *Rikenella* and *Corynebacterium_1*, distinguished the normal and model groups. Figure 11B,C shows that *Escherichia-Shigella*, *Aerococcus*, *Staphylococcus*, and *Proteus* are characteristic taxa in SPI versus model comparisons, while *Psychrobacter*, *Escherichia-Shigella,* and *Aerococcus* were distinctive for 20% HSP versus model comparisons. In addition, *Psychrobacter*, *Proteus*, and *Staphylococcus* differentiated the SPI group and the 20% HSP group.

It was reported that *Rikenella* may influence glucose and lipid metabolism [65], the immune system [66], and intestinal function [67]. In general, *Rikenella* has been considered to be a potential probiotic, while its abundance may increase during immune deficiencies. Studies have shown that *Rikenella* could be enriched in innate immune system deficiency mice, accompanied by elevated levels of *Lactobacillus* [66], which is a beneficial bacterium. This was consistent with the results of our experiment in which the abundance of *Rikenella* and *Lactobacillus* increased in the model group, indicating that immunosuppression may selectively promote the proliferation of certain intestinal probiotics. Meanwhile, SPI and 20% HSP intake inhibited the proliferation of these two bacteria, which further indicated that the intestinal microbiota composition could be influenced by immune function.

As typical intestinal bacteria, *Escherichia-Shigella* were dominant in the normal group and model group, while their abundance decreased sharply in the SPI group and 20% HSP group. It might be that the intake of SPI and 20% HSP alter the microbiota, inhibiting the growth of *Escherichia-Shigella*, which is negatively correlated with immunity [68].

*Proteus*, which is widespread in the environment and comprises part of the normal intestinal microbiota, is typically considered an opportunistic pathogen. However, it emerged as a key differential genus between the 20% HSP group and SPI group, with significantly increased abundance in the former. This *Proteus* enrichment may represent a non-pathogenic response, as studies reported that *Proteus* exhibited anti-microbial activity against *C. albicans*, *Bacillus subtilis*, and *Escherichia coli* [69]. This antimicrobial function may partially explain the inhibition of *Escherichia-Shigella* observed in the 20% HSP group.

*Psychrobacter* are Gram-negative bacteria belonging to the *Moraxellaceae* family. Although some species are associated with human and animal diseases, they possess efficient iron acquisition mechanisms and exhibit broad resistance to clinical antimicrobials [70]. Few studies address *Psychrobacter*’s impact on intestinal microbiota, leaving its direct relationship with intestinal and immune functions uncertain. Notably, research suggests increased *Psychrobacter* abundance may benefit microbiota regulation: lactoferrin modified gut microbial structures in obese mice, elevating *Alistipes*, *Acidobacteriota*, and *Psychrobacter* populations [71].

Chronic CTX-induced immunosuppression altered gut microbiota composition. SPI and 20% HSP interventions counteracted these changes by suppressing *Escherichia-Shigella* and modulating *Proteus* and *Psychrobacter* in 20% HSP-treated mice. These coordinated shifts demonstrate microbiota–immune crosstalk and highlight HSP/SPI’s therapeutic potential in restoring gut homeostasis.

## 4. Conclusions

In this study, we investigated the potential mechanism of SPI and 20% HSP using *C. elegans* as a model organism, examining basic physiological indices, antioxidant capacity, oxidative stress resistance, lipofuscin content, and the expression levels of ageing-related genes. The results showed that SPI and 20% HSP could significantly enhance nematodes’ antioxidant capacity and reduce age-associated lipofuscin accumulation in nematodes. Subsequent qRT-PCR analysis revealed SPI and 20% HSP’s modulation of longevity pathways, confirming critical roles for transcription factors *daf-2* and *daf-16* in nematode lifespan regulation.

Meanwhile, it was indicated that continuous low-dose CTX injection could induce long-term suppression of immune function in mice, rather than high-dose injection administered once or several times. This suppression was reflected in the reduction of body weight, the decrease in WBC and lymph in the blood, the atrophy of the thymus, and the damage to spleen and thymus tissues. Compared with SPI, 20% HSP showed stronger effects on the recovery of these immune indicators. The investigation on liver antioxidant activity showed that 20% HSP had a significant reduction effect on the peroxide product MDA. Meanwhile, CTX might not induce severe inflammations or infections, and neither SPI nor 20% HSP increased cytokines to a normal level, indicating they had no significant effect on inflammation in vivo as dietary proteins. In addition, SPI and 20% HSP could cause changes in the intestinal microbiota of mice, and these changes were significantly different between the two groups. This suggested that SPI and 20% HSP or their digestive products and metabolites may have distinct effects on colon microbes.

In summary, as the hydrolysis product of SPI, 20% HSP exhibited a pretty good protective effect on oxidative stress and immune functions, especially on the long-term suppression of immune function in tested animals induced by CTX in this study. Furthermore, the regulatory effect of 20% HSP on intestinal microbiota may reflect its role in promoting overall health, which needs to be further explored around the intestinal functional axis in the future.

## Data Availability

The original contributions presented in the study are included in the article, further inquiries can be directed to the corresponding author.

## Supplementary Materials

The following supporting information can be downloaded at: https://www.mdpi.com/article/10.3390/antiox14060689/s1, Figure S1: Electrophoretic analysis of hydrolyzed soybean protein isolate; Figure S2: Heatmap of colonic microbiota at the genus level; Table S1: Primer sequences for the genes involved in anti-aging in *C. elegans*; Table S2: Primer information for 16S rDNA.

## Abbreviations

The following abbreviations are used in this manuscript:

SPI	Soybean protein isolate
HSP	Hydrolyzed soybean protein
*C. elegans*	*Caenorhabditis elegans*
CTX	Cyclophosphamide

## References

[1] Navarro-Hortal M.D., Romero-Márquez J.M., López-Bascón M.A., Sánchez-González C., Xiao J., Sumalla-Cano S., Battino M., Forbes-Hernández T.Y., Quiles J.L. (2024). In Vitro and In Vivo Insights into a Broccoli Byproduct as a Healthy Ingredient for the Management of Alzheimer’s Disease and Aging through Redox Biology. J. Agric. Food Chem..

[2] Karabulut G., Subasi B.G., Ivanova P., Goksen G., Chalova V., Capanoglu E. (2025). Towards sustainable and nutritional-based plant protein sources: A review on the role of rapeseed. Food Res. Int..

[3] Noreen S., Tufail T., Ul Ain H.B., Awuchi C.G. (2023). Pharmacological, nutraceutical, and nutritional properties of flaxseed (*Linum usitatissimum*): An insight into its functionality and disease mitigation. Food Sci. Nutr..

[4] Ullah H., De Filippis A., Baldi A., Dacrema M., Esposito C., Garzarella E.U., Santarcangelo C., Tantipongpiradet A., Daglia M. (2021). Beneficial Effects of Plant Extracts and Bioactive Food Components in Childhood Supplementation. Nutrients.

[5] Navarro-Hortal M.D., Romero-Márquez J.M., Jiménez-Trigo V., Xiao J., Giampieri F., Forbes-Hernández T.Y., Grosso G., Battino M., Sánchez-González C., Quiles J.L. (2023). Molecular bases for the use of functional foods in the management of healthy aging: Berries, curcumin, virgin olive oil and honey; three realities and a promise. Crit. Rev. Food Sci. Nutr..

[6] Ijinu T.P., De Lellis L.F., Shanmugarama S., Pérez-Gregorio R., Sasikumar P., Ullah H., Buccato D.G., Di Minno A., Baldi A., Daglia M. (2023). Anthocyanins as Immunomodulatory Dietary Supplements: A Nutraceutical Perspective and Micro-/Nano-Strategies for Enhanced Bioavailability. Nutrients.

[7] Grosso G., Godos J., Currenti W., Micek A., Falzone L., Libra M., Giampieri F., Forbes-Hernández T.Y., Quiles J.L., Battino M. (2022). The Effect of Dietary Polyphenols on Vascular Health and Hypertension: Current Evidence and Mechanisms of Action. Nutrients.

[8] Wang L., Li P., Zheng F., Zhu Z., Bai F., Gao R. (2024). Collagen peptides from sturgeon swim bladder prolong the lifespan and healthspan in *Caenorhabditis elegans*. J. Sci. Food Agric..

[9] Chen M.L., Zhu X.H., Ran L., Lang H.D., Yi L., Mi M.T. (2017). Trimethylamine-N-Oxide Induces Vascular Inflammation by Activating the NLRP3 Inflammasome Through the SIRT3-SOD2-mtROS Signaling Pathway. J. Am. Heart Assoc..

[10] Zhang Y., Hao R., Chen J., Li S., Huang K., Cao H., Farag M.A., Battino M., Daglia M., Capanoglu E. (2024). Health benefits of saponins and its mechanisms: Perspectives from absorption, metabolism, and interaction with gut. Crit. Rev. Food Sci. Nutr..

[11] Gao X., Liu E., Zhang J., Yang M., Chen S., Liu Z., Ma H., Hu F. (2019). Effects of sonication during moromi fermentation on antioxidant activities of compounds in raw soy sauce. LWT.

[12] Xia T., Duan W., Zhang Z., Fang B., Zhang B., Xu B., de la Cruz C.B.V., El-Seedi H., Simal-Gandara J., Wang S. (2021). Polyphenol-rich extract of Zhenjiang aromatic vinegar ameliorates high glucose-induced insulin resistance by regulating JNK-IRS-1 and PI3K/Akt signaling pathways. Food Chem..

[13] Kusumah J., Gonzalez de Mejia E. (2022). Impact of soybean bioactive compounds as response to diet-induced chronic inflammation: A systematic review. Food Res. Int..

[14] Peng C., Wang X., Chen J., Jiao R., Wang L., Li Y.M., Zuo Y., Liu Y., Lei L., Ma K.Y. (2014). Biology of ageing and role of dietary antioxidants. Biomed. Res. Int..

[15] Kim I.S., Kim C.H., Yang W.S. (2021). Physiologically Active Molecules and Functional Properties of Soybeans in Human Health-A Current Perspective. Int. J. Mol. Sci..

[16] Wen L., Jiang Y., Zhou X., Bi H., Yang B. (2021). Structure identification of soybean peptides and their immunomodulatory activity. Food Chem..

[17] Rebholz C.M., Reynolds K., Wofford M.R., Chen J., Kelly T.N., Mei H., Whelton P.K., He J. (2013). Effect of soybean protein on novel cardiovascular disease risk factors: A randomized controlled trial. Eur. J. Clin. Nutr..

[18] Draganidis D., Karagounis L.G., Athanailidis I., Chatzinikolaou A., Jamurtas A.Z., Fatouros I.G. (2016). Inflammaging and skeletal muscle: Can protein intake make a difference?. J. Nutr..

[19] Chalamaiah M., Yu W., Wu J. (2018). Immunomodulatory and anticancer protein hydrolysates (peptides) from food proteins: A review. Food Chem..

[20] Dai C., Yan P., Xu X., Huang L., Dabbour M., Benjamin K.M., He R., Ma H. (2023). Effect of single and two-stage fermentation on the antioxidative activity of soybean meal, and the structural and interfacial characteristics of its protein. LWT.

[21] Li L., Yang Y., Ma C.-M., Wang B., Bian X., Zhang G., Liu X.-F., Zhang N. (2025). Structure, antioxidant activity, and neuroprotective effect of black soybean (*Glycine max* (L.) merr.) protein hydrolysates. Food Chem..

[22] Li T., Zhang X., Ren Y., Zeng Y., Huang Q., Wang C. (2022). Antihypertensive effect of soybean bioactive peptides: A review. Curr. Opin. Pharmacol..

[23] Wang W., De Mejia E.G. (2005). A New Frontier in Soy Bioactive Peptides that May Prevent Age-related Chronic Diseases. Compr. Rev. Food Sci. F.

[24] Singh B.P., Aluko R.E., Hati S., Solanki D. (2022). Bioactive peptides in the management of lifestyle-related diseases: Current trends and future perspectives. Crit. Rev. Food Sci. Nutr..

[25] Ma H., Liu R., Zhao Z., Zhang Z., Cao Y., Ma Y., Guo Y., Xu L. (2016). A Novel Peptide from Soybean Protein Isolate Significantly Enhances Resistance of the Organism under Oxidative Stress. PLoS ONE.

[26] Singh B.P., Vij S., Hati S. (2014). Functional significance of bioactive peptides derived from soybean. Peptides.

[27] Zaky A.A., Simal-Gandara J., Eun J.-B., Shim J.-H., Abd El-Aty A.M. (2022). Bioactivities, applications, safety, and health benefits of bioactive peptides from food and by-products: A review. Front. Nutr..

[28] Yan Z., Gui Y., Liu C., Zhang X., Wen C., Olatunji O.J., Suttikhana I., Ashaolu T.J. (2024). Gastrointestinal digestion of food proteins: Anticancer, antihypertensive, anti-obesity, and immunomodulatory mechanisms of the derived peptides. Food Res. Int..

[29] Wong F.-C., Xiao J., Wang S., Ee K.-Y., Chai T.-T. (2020). Advances on the antioxidant peptides from edible plant sources. Trends Food Sci. Tech..

[30] Shadrack S.M., Wang Y., Mi S., Lu R., Zhu Y., Tang Z., McClements D.J., Cao C., Xu X., Li W. (2025). Enhancing bioavailability and functionality of plant peptides and proteins: A review of novel strategies for food and pharmaceutical applications. Food Chem..

[31] Markaki M., Tavernarakis N. (2020). *Caenorhabditis elegans* as a model system for human diseases. Curr. Opin. Biotechnol..

[32] Ley R.E., Turnbaugh P.J., Klein S., Gordon J.I. (2006). Microbial ecology: Human gut microbes associated with obesity. Nature.

[33] Jiang Q., Charoensiddhi S., Xue X., Sun B., Liu Y., El-Seedi H.R., Wang K. (2023). A review on the gastrointestinal protective effects of tropical fruit polyphenols. Crit. Rev. Food Sci. Nutr..

[34] Bai J., Zhu Y., Dong Y. (2018). Modulation of gut microbiota and gut-generated metabolites by bitter melon results in improvement in the metabolic status in high fat diet-induced obese rats. J. Funct. Foods.

[35] Wu S., Bhat Z.F., Gounder R.S., Mohamed Ahmed I.A., Al-Juhaimi F.Y., Ding Y., Bekhit A.E.D.A. (2022). Effect of Dietary Protein and Processing on Gut Microbiota-A Systematic Review. Nutrients.

[36] Liu R., Qu L., Niu X., Li S., Li C., Liu J. (2018). Study of the Relationship between the Structure and Function Properties on Soy Protein Isolate at Different Hydrolysis Degree. Food Res. Dev..

[37] Rutherfurd S.M. (2010). Methodology for determining degree of hydrolysis of proteins in Hydrolysates: A review. J. AOAC Int..

[38] Bai J., Li J., Pan R., Zhu Y., Xiao X., Li Y., Li C. (2021). Polysaccharides from Volvariella volvacea inhibit fat accumulation in *C. elegans* dependent on the *aak-2/nhr-49*-mediated pathway. J. Food Biochem..

[39] Zhao Y., Xiao M., Eweys A.S., Bai J., Darwesh O.M., Xiao X. (2023). Cinnamaldehyde Alleviates the Oxidative Stress of *Caenorhabditis elegans* in the Presence of Lactic Acid. Plant Foods Hum. Nutr..

[40] Chong J., Liu P., Zhou G., Xia J. (2020). Using MicrobiomeAnalyst for comprehensive statistical, functional, and meta-analysis of microbiome data. Nat. Protoc..

[41] Liu J.Y., Zheng R.Q., Wang Y., Liu Y.H., Jiang S., Wang X.Z., He K., Pan X., Zhou T., Li T. (2022). The Endogenous Metabolite Glycerophosphocholine Promotes Longevity and Fitness in *Caenorhabditis elegans*. Metabolites.

[42] Lin Q., Song B., Zhong Y., Yin H., Li Z., Wang Z., Cheong K.L., Huang R., Zhong S. (2023). Effect of Sodium Hyaluronate on Antioxidant and Anti-Ageing Activities in *Caenorhabditis elegans*. Foods.

[43] Yu X., Li H., Lin D., Guo W., Xu Z., Wang L., Guan S. (2021). Ginsenoside Prolongs the Lifespan of *C.* elegans via Lipid Metabolism and Activating the Stress Response Signaling Pathway. Int. J. Mol. Sci..

[44] Kritikos S., Papanikolaou K., Draganidis D., Poulios A., Georgakouli K., Tsimeas P., Tzatzakis T., Batsilas D., Batrakoulis A., Deli C.K. (2021). Effect of whey vs. soy protein supplementation on recovery kinetics following speed endurance training in competitive male soccer players: A randomized controlled trial. J. Int. Soc. Sports Nutr..

[45] Goleij P., Khandan M., Khazeei Tabari M.A., Sanaye P.M., Alijanzadeh D., Soltani A., Hosseini Z., Larsen D.S., Khan H., Kumar A.P. (2025). Unlocking the Potential: How Flavonoids Affect Angiogenesis, Oxidative Stress, Inflammation, Proliferation, Invasion, and Alter Receptor Interactions in Endometriosis. Food Sci. Nutr..

[46] Finkel T., Holbrook N.J. (2000). Oxidants, oxidative stress and the biology of ageing. Nature.

[47] Mazzoni L., Giampieri F., Alvarez Suarez J.M., Gasparrini M., Mezzetti B., Forbes Hernandez T.Y., Battino M.A. (2019). Isolation of strawberry anthocyanin-rich fractions and their mechanisms of action against murine breast cancer cell lines. Food Funct..

[48] Wang Q., Wu J., Huang J., Yang L., Tao J., Nie J., Zhao J., Wang Y.N. (2023). *Cremastra appendiculata* polysaccharides improve stress resistance and prolong the lifespan of *Caenorhabditis elegans* via *daf-16* in the insulin signaling pathway. Int. J. Biol. Macromol..

[49] Gusarov I., Pani B., Gautier L., Smolentseva O., Eremina S., Shamovsky I., Katkova-Zhukotskaya O., Mironov A., Nudler E. (2017). Glycogen controls *Caenorhabditis elegans* lifespan and resistance to oxidative stress. Nat. Commun..

[50] Fan Y., Wang M., Li Z., Jiang H., Shi J., Shi X., Liu S., Zhao J., Kong L., Zhang W. (2022). Intake of Soy, Soy Isoflavones and Soy Protein and Risk of Cancer Incidence and Mortality. Front. Nutr..

[51] Westfall S., Lomis N., Prakash S. (2018). A novel polyphenolic prebiotic and probiotic formulation have synergistic effects on the gut microbiota influencing Drosophila melanogaster physiology. Artif. Cell Nanomed. B.

[52] Xiao C.W. (2008). Health effects of soy protein and isoflavones in humans. J. Nutr..

[53] Zhao J., Yu J., Zhi Q., Yuan T., Lei X., Zeng K., Ming J. (2021). Anti-aging effects of the fermented anthocyanin extracts of purple sweet potato on *Caenorhabditis elegans*. Food Funct..

[54] Song B., Zheng B., Li T., Liu R.H. (2020). Raspberry extract promoted longevity and stress tolerance via the insulin/IGF signaling pathway and DAF-16 in *Caenorhabditis elegans*. Food Funct..

[55] Zhou Y., Xu Q., Zhou X., Song S., Zhu B. (2018). Stress resistance and lifespan extension of *Caenorhabditis elegans* enhanced by peptides from mussel *(Mytilus edulis*) protein hydrolyzate. Food Funct..

[56] Desaka N., Nishikawa H., Honda Y., Matsumoto K., Matsuzaki C., Mizushima K., Takagi T., Naito Y., Higashimura Y. (2022). Oligosaccharides from agar extends lifespan through activation of unfolded protein response via *SIR-2.1* in *Caenorhabditis elegans*. Eur. J. Nutr..

[57] Zečić A., Braeckman B.P. (2020). DAF-16/FoxO in *Caenorhabditis elegans* and Its Role in Metabolic Remodeling. Cells.

[58] Shen P., Yue Y., Park Y. (2018). A living model for obesity and aging research: *Caenorhabditis elegans*. Crit. Rev. Food Sci. Nutr..

[59] Kimura K.D., Tissenbaum H.A., Liu Y., Ruvkun G. (1997). *daf-2*, an insulin receptor-like gene that regulates longevity and diapause in *Caenorhabditis elegans*. Science.

[60] Kenyon C., Chang J., Gensch E., Rudner A., Tabtiang R.A.C. (1993). *elegans* mutant that lives twice as long as wild type. Nature.

[61] Gómez-Gallego C., Frias R., Pérez-Martínez G., Bernal M.J., Periago M.J., Salminen S., Ros G., Collado M.C. (2014). Polyamine supplementation in infant formula: Influence on lymphocyte populations and immune system-related gene expression in a Balb/cOlaHsd mouse model. Food Res. Int..

[62] Zhang H., Yu R., Liu X., Guo X., Zeng Z. (2012). The expression of PAC1 increases in the degenerative thymus and low dose PACAP protects female mice from cyclophosphamide induced thymus atrophy. Peptides.

[63] Adams A., De Kimpe N., van Boekel M.A.J.S. (2008). Modification of casein by the lipid oxidation product malondialdehyde. J. Agric. Food Chem..

[64] Gao R., Qi Z., Lin J., Wang G., Chen G., Yuan L., Sun Q. (2023). Chondroitin Sulfate Alleviated Obesity by Modulating Gut Microbiota and Liver Metabolome in High-Fat-Diet-Induced Obese Mice. J. Agric. Food Chem..

[65] Luo Y., Wen Y., Huang J., Chen B., Lv S., Qiu H., Li S., Liu S., Yang Q., He L. (2024). Matcha alleviates obesity by modulating gut microbiota and its metabolites. Curr. Res. Food Sci..

[66] Wu M., Wang F., Yang J., Li P., Yan D., Yang Y., Zhang W., Ren J., Zhang Z., Wang M. (2020). The responses of the gut microbiota to MBL deficiency. Mol. Immunol..

[67] Sun J., Liu J., Ren G., Chen X., Cai H., Hong J., Kan J., Jin C., Niu F., Zhang W. (2022). Impact of purple sweet potato (*Ipomoea batatas* L.) polysaccharides on the fecal metabolome in a murine colitis model. RSC Adv..

[68] Zhang Q., Tang X., Zhang X., He Y., Li Y. (2023). Immunomodulatory effect of two hydrolysates of chitin on cyclophosphamide-induced mice via immune response enhancement and gut microbiota modulation. J. Funct. Foods.

[69] Graça A.P., Viana F., Bondoso J., Correia M.I., Gomes L., Humanes M., Reis A., Xavier J.R., Gaspar H., Lage O.M. (2015). The antimicrobial activity of heterotrophic bacteria isolated from the marine sponge Erylus deficiens (Astrophorida, Geodiidae). Front Microbiol..

[70] García-López M.L., Santos J.A., Otero A., Rodríguez-Calleja J.M., Batt C.A., Tortorello M.L. (2014). Psychrobacter. Encyclopedia of Food Microbiology.

[71] Wang W., Zhang J., Li Y., Su S., Wei L., Li L., Hu R. (2024). Lactoferrin alleviates chronic low-grade inflammation response in obese mice by regulating intestinal flora. Mol. Med. Rep..

