# Supplementary material for: A Hydrolyzed Soybean Protein Enhances Oxidative Stress Resistance in C. elegans and Modulates Gut–Immune Axis in BALB/c Mice
Source: Antioxidants (Basel). 2025 Jun 5;14(6):689. doi: 10.3390/antiox14060689 (PMC12189443; doi:10.3390/antiox14060689)
Supplement: Supplementary file 1 [file antioxidants-14-00689-s001.zip › antioxidants-3626338-supplementary.pdf]

Supplementary Table

Table S1. Primer sequences for the genes involved in anti-aging in *C. elegans*

Genes	Forward sequences (5'-3')	Reverse sequences (5'-3')
daf-16	CCAGACGGAAGGCTTAAACT	ATTCGCATGAAACGAGAATG
daf-2	GGATAAAGGCGAATCAAAGTGTC	CGATACACTTTCCCTTGTGATAGAC
skn-1	AGTGTCGGCGTTCCAGATTTC	GTCGACGAATCTTGCGAATCA
hsf-16.2	GAATGCGACTAGGCAAATGGC	GGTGGATGAGGTGGAAGTCG
akt-1	TGAATCCAACGCTGACGAAC	AAAACATTCAGCGAAGCACG
act-1	TCGGTATGGGACAGAAGGAC	CATCCCAGTTGGTGACGATA

Table S2. Primer information for 16S rDNA

Region	Primer name	Primer sequence	Product length
V4	515F	GTGYCAGCMGCCGCGGTAA	~292
	806R	GGACTACNVGGGTWTCTAAT	
V3-V4	341F	CCTACGGGNGGCWGCAG	~466
	806R	GGACTACHVGGGTATCTAAT	
V5-V7	799F	AACMGGATTAGATACCCKG	~412
	1193R	ACGTCATCCCCACCTTCC	
V4-V5	515F	GTGCCAGCMGCCGCGGTAA	~414
	907R	CCGTCAATTCCTTTGAGTTT	
V4-V5	Arch519F	CAGCMGCCGCGGTAA	~416
	Arch915R	GTGCTCCCCCGCCAATTCCT	

Supplementary Figures

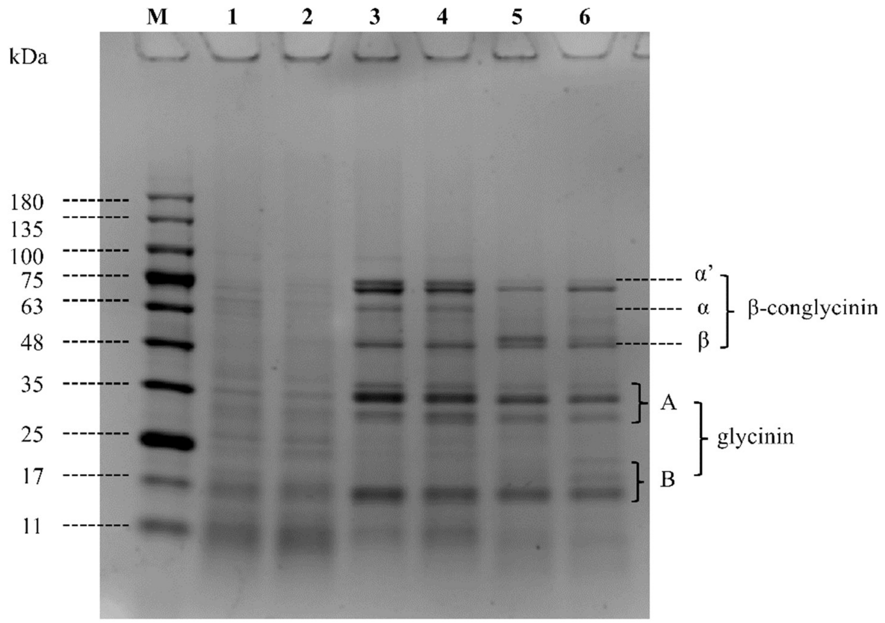

M：marker, 1: 20% degree of hydrolysis (DH), 2: 27% DH, 3: 16.2% DH, 4: 10% DH, 5: 31% DH, 6:38.9% DH

Figure S1. Electrophoretic analysis of hydrolyzed soybean protein isolate

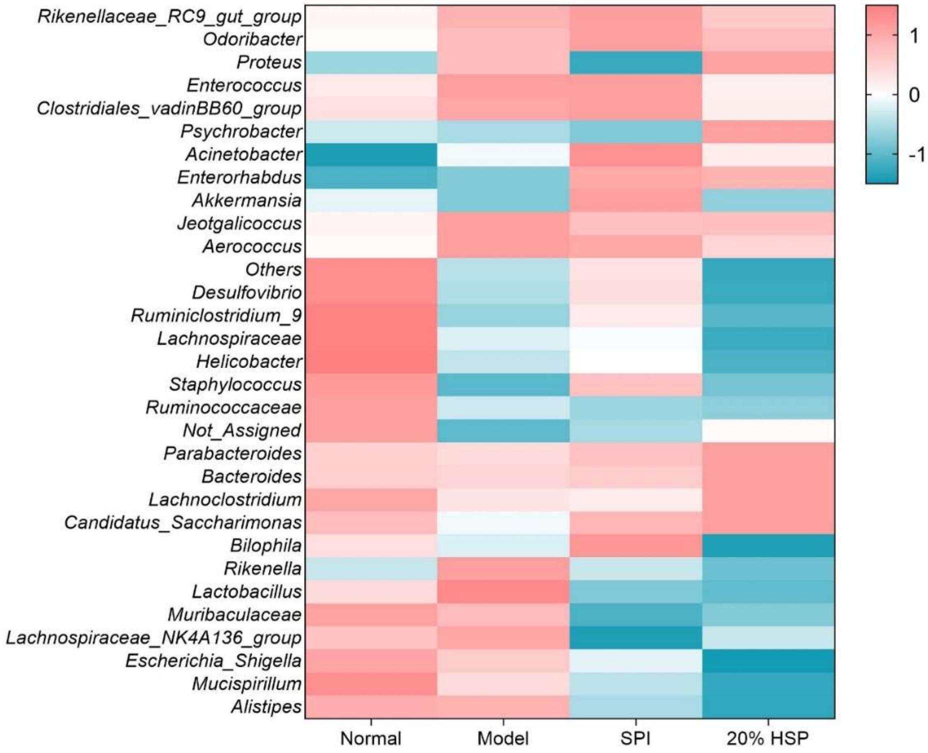

Figure S2. Heatmap of colonic flora at genus level
